# Prognostic performance of pre-treatment NLR and PLR in patients suffering from osteosarcoma

**DOI:** 10.1186/s12957-016-0889-2

**Published:** 2016-04-29

**Authors:** Wen-Kai Xia, Zhi-Li Liu, Dong Shen, Qing-Feng Lin, Jun Su, Wei-Dong Mao

**Affiliations:** Department of Nephrology, The Affiliated Jiangyin Hospital of Southeast University Medical College, Jiangyin, Jiangsu 214400 China; Department of Oncology, The Affiliated Jiangyin Hospital of Southeast University Medical College, 163 Shoushan Road, Jiangyin, Jiangsu 214400 China

**Keywords:** Osteosarcoma, NLR, PLR, Prognosis

## Abstract

**Background:**

Inflammatory response markers have been proposed to predict the clinical outcomes in various cancers. The aim of this study was to explore the influence of the neutrophil-to-lymphocyte ratio (NLR) and platelet-to-lymphocyte ratio (PLR) on the prognosis of osteosarcoma.

**Methods:**

Three hundred fifty-nine patients who underwent curative surgery for osteosarcoma were enrolled from 2005 to 2010. NLR and PLR were calculated from peripheral blood cell counts taken at pre-treatment. Optimal cutoff values of NLR and PLR were determined on the basis of receiver operating characteristic curve analysis. A predictive model was established to predict the clinical outcome for overall survival, and the predictive accuracy of this model was determined by concordance index (*c*-index).

**Results:**

Our results showed that advanced stage and metastasis at diagnosis were significantly associated with the high NLR and PLR groups. NLR was an independent prognostic indicator for overall survival (HR = 1.80, 95 % CI = 1.35–2.41, *P* < 0.001) and progression-free survival (HR = 1.65, 95 % CI = 1.26–2.15, *P* < 0.001), except for PLR. The nomogram could perform well in the prediction of overall survival in patients with osteosarcoma (*c*-index 0.829).

**Conclusions:**

Our results suggest that both NLR and PLR can reflect clinical prognosis. NLR is more predictive of overall survival and progression-free survival than PLR.

## Background

Osteosarcoma is one of the most frequent malignant tumors of the bone [1]. Two peaks of incidence appear in osteosarcoma patients, such as children and early adolescence aged 15 to 19 years [2]. Patients’ age is increasing [3], which the number of patients over 40 years old account for 13–30 % of osteosarcoma patients [4]. Due to its rapid progression and poor prognosis, osteosarcoma is a major death-causing disease [5]. Despite substantial progress achieved in the diagnosis and treatment for osteosarcoma in the past decades, its clinical outcome remains unsatisfactory due to local relapse or metastasis after resection of primary osteosarcoma.

The poor prognosis of osteosarcoma partially results from lack of a better biomarker to detect it at an early tumor stage. The ability to predict the precise prognosis of a patient is indispensible for selecting the appropriate treatment plan and follow-up strategies. Although prognostic indicators have the Enneking surgical criteria [6] and alkaline phosphatase, heterogeneous clinical outcomes are frequently observed within the same tumor stage. Therefore, it is necessary for us to further understand underlying mechanisms and find a useful biomarker of osteosarcoma to predict clinical outcome.

Recently, emerging evidence revealed that systemic inflammatory response has been reported to be an independent prognostic biomarker in various types of tumors. Published literatures have shown a significant link between inflammatory markers and poor survival in several types of tumors, including thrombocytosis, leukocytosis, high neutrophil-to-lymphocyte ratio (NLR), or platelet-to-lymphocyte ratio (PLR) [7–11]. However, the influence of NLR and PLR on the prognosis of osteosarcoma patients has been not reported. Herein, the purpose of this study was to evaluate the influence of NLR and PLR on clinical prognosis in 359 patients with osteosarcoma after curative surgery.

## Methods

### Patients

The Medical Ethics Committee of The Affiliated Jiangyin Hospital, School of Medicine, Southeast University approved this study. Informed consent has been obtained from each patient. Medical records of all newly diagnosed osteosarcoma patients between 2005 and 2010 in The Affiliated Jiangyin Hospital, School of Medicine, Southeast University were reviewed and retrospectively analyzed in the present study. The diagnosis of osteosarcoma was confirmed depended on histological evidence and classified based on the Enneking surgical criteria [6]. The inclusion criteria were as follows. (1) All patients with osteosarcoma underwent a corresponding appropriate treatment according to standard therapeutic strategies. (2) Inflammatory markers were obtained prior to anticancer treatment, such as surgery, blood transfusion, chemotherapy, and radiotherapy. (3) No hematology disease, infection, and hyperpyrexia. Finally, 359 patients were enrolled in the present study. Clinical features of eligible patients were collected including age, sex, tumor location, pathological fracture, tumor size, alkaline phosphatase (ALP), clinical stage, metastasis at diagnosis, and post-operative chemotherapy.

### Blood sample analysis

Blood samples were obtained before treatment for the measurement of the white cell, neutrophil, lymphocyte, and platelet counts.

### Definition and optimal cutoff values of NLR and PLR

NLR was defined as the neutrophil counts divided by the lymphocyte counts. PLR was defined as the platelet counts divided by the lymphocyte counts. Using overall survival and progression-free survival, respectively, as end points, optimal cutoff values of NLR and PLR were obtained when the Youden index was maximal. Subsequently, patients with a NLR or PLR greater than the corresponding cutoff values were defined as high NLR or PLR (HNLR or HPLR), and others were defined as low NLR or PLR (LNLR or LPLR).

### Patient follow-up

Each patient was followed up regularly until death or December 2014 at post-operation (every 3 months for the first 2 years and then every 6 months up to the 5th year). Physical examination, laboratory tests, and imageological diagnosis were performed at every visit. The follow-up period varied from 3 months to 5 years, with a median of 40 months. Overall survival (OS) was calculated from treatment to death. The date of last follow-up was used for dropout patients. Progression-free survival (PFS) was collected from treatment to disease progression or relapse or until the date of the last follow-up.

### Statistical analysis

To evaluate the sensitivity and specificity for the 5-year OS and PFS, the receiver operating characteristic (ROC) curve was applied, and the largest Youden’s index was estimated to determine the optimal NLR and PLR cutoff values. Comparison of categorical variables was conducted using a chi-square test. Comparison of continuous variables was performed using Mann-Whitney *U* or Kruskal-Wallis test. Survival curves were plotted by the Kaplan-Meier method, and the significance was assessed by the log-rank test. The significant predictors for OS and PFS determined by univariate analysis were evaluated by multivariate analysis using Cox’s proportional hazards model. Nomogram for OS was constructed by R 3.0.3 software (Institute for Statistics and Mathematics, Austria), and Harrell’s concordance index (*c*-index) was used to evaluate the predictive accuracy. All results analyses were performed by SPSS 20.0 software (IBM, USA). *P* < 0.05 was considered statistically significant.

## Results

### Clinicopathological characteristics

Of 359 patients with osteosarcoma, 258 (71.87 %) were male, and the median age was 50 years (range 19–69 years; Table 1); 62 (17.27 %) patients with pathological fracture and 132 (36.77 %) patients with initial metastasis were recorded from newly diagnosed patients. According to Enneking surgical staging criteria, the number of stages I–II and III was 161 (44.85 %) and 198 (55.15 %), respectively. Pathological results suggested that 290 (80.78 %) patients’ osteosarcomas were located in the tibia or femur, and tumor size was more than 8 cm in 158 (44.01 %) patients with osteosarcoma. During the follow-up period, 187 (50.09 %) patients had experienced chemotherapy. Among all enrolled patients, 211 (58.77 %) patients died from cancer-related disease; 249 (69.40 %) patients were detected as local recurrence or metastasis. The median values of the pre-operative white blood cells, neutrophil, lymphocyte, and platelet counts were 6.20 × 10^9^/L, 4.16 × 10^9^/L, 1.24 × 10^9^/L, and 183 × 10^9^/L, respectively. The median values of NLR and PLR were 3.19 and 142, respectively.

**Table 1:** Association of the patients’ clinical parameters with NLR and PLR

Clinical parameters	Total	NLR		*P*	PLR		*P*
		Low NLR	High NLR		Low PLR	High PLR	
	*n* = 359 (%)	*n* = 195 (%)	*n* = 164 (%)		*n* = 136 (%)	*n* = 223 (%)	
Age (year)^a^	48.7 ± 12.4	48.5 ± 11.5	49.0 ± 13.4	0.536	46.4 ± 12.3	50.2 ± 12.3	0.006
Sex							
Male	258 (71.87 %)	137 (70.26 %)	121 (73.78 %)	0.459	92 (67.65 %)	166 (74.44 %)	0.165
Female	101 (28.13 %)	58 (29.74 %)	43 (26.22 %)		44 (32.35 %)	57 (25.56 %)	
Tumor location							
Tibia/femur	290 (80.78 %)	156 (80.00 %)	134 (81.71 %)	0.683	104 (76.47 %)	186 (83.41 %)	0.106
Elsewhere	69 (19.22 %)	39 (20.00 %)	30 (18.29 %)		32 (23.53 %)	37 (16.59 %)	
Pathological fracture							
Yes	62 (17.27 %)	37 (18.97 %)	25 (15.24 %)	0.352	25 (18.38 %)	37 (16.59 %)	0.663
No	297 (82.73 %)	158 (81.03 %)	139 (84.76 %)		111 (81.62 %)	186 (83.41 %)	
Tumor size							
<8 cm	201 (55.99 %)	110 (56.41 %)	91 (55.49 %)	0.861	76 (55.88 %)	125 (56.05 %)	0.975
≥8 cm	158 (44.01 %)	85 (43.59 %)	73 (44.51 %)		60 (44.11 %)	98 (43.95 %)	
ALP							
Elevated	104 (28.97 %)	48 (24.62 %)	56 (34.15 %)	0.047	35 (25.74 %)	69 (30.94 %)	0.291
Normal	255 (71.03 %)	147 (75.38 %)	108 (65.85 %)		101 (74.26)	154 (69.06 %)	
Clinical stage^b^							
I–II	161 (44.85 %)	111 (56.92 %)	50 (30.49 %)	0.000	73 (53.68 %)	88 (39.46 %)	0.009
III	198 (55.15 %)	84 (43.08 %)	114 (69.51 %)		63 (46.32 %)	135 (60.54 %)	
Metastasis at diagnosis							
Present	132 (36.77 %)	41 (21.03 %)	91 (55.49 %)	0.000	33 (24.26 %)	99 (44.39 %)	0.000
Absent	227 (63.23 %)	154 (78.97 %)	73 (44.51 %)		103 (75.74 %)	124 (55.61 %)	
Post-chemotherapy							
Yes	187 (50.09 %)	95 (48.72 %)	92 (56.10 %)	0.163	67 (49.26 %)	120 (53.81 %)	0.403
No	172 (47.91 %)	100 (51.28 %)	72 (43.90 %)		69 (50.74 %)	103 (46.19 %)	
WBC counts (*10^9^/L)^c^	6.20 (1.90–26.68)	5.04 (1.90–13.00)	8.01 (3.10–26.68)	0.000	5.71 (1.90–24.02)	6.64 (2.20–26.68)	0.027
Neutrophil counts (*10^9^/L)^c^	4.16 (0.84–26.07)	2.91 (0.84–7.40)	6.50 (2.42–26.07)	0.000	3.47 (0.84–21.93)	4.88 (1.10–26.07)	0.000
Lymphocyte counts (*10^9^/L)^c^	1.24 (0.09–4.84)	1.59 (0.61–4.84)	0.91 (0.09–4.46)	0.000	1.62 (0.54–4.84)	1.06 (0.09–2.62)	0.000
Platelet counts (*10^9^/L)^c^	183 (18–516)	183 (26–516)	175 (18–490)	0.890	141 (26–292)	225 (18–516)	0.000
NLR	3.19 (0.79–74.49)						
PLR	142 (5.4–3111)						

*ALP* alkaline phosphatase, *WBC* white blood cell, *NLR* neutrophil-to-lymphocyte ratio, *PLR* platelet-to-lymphocyte ratio

^a^Mean ± SD

^b^Clinical stage according to Enneking surgical stage

^c^Median (range)

### The optimal cutoff values for NLR and PLR

**Fig. 1:**
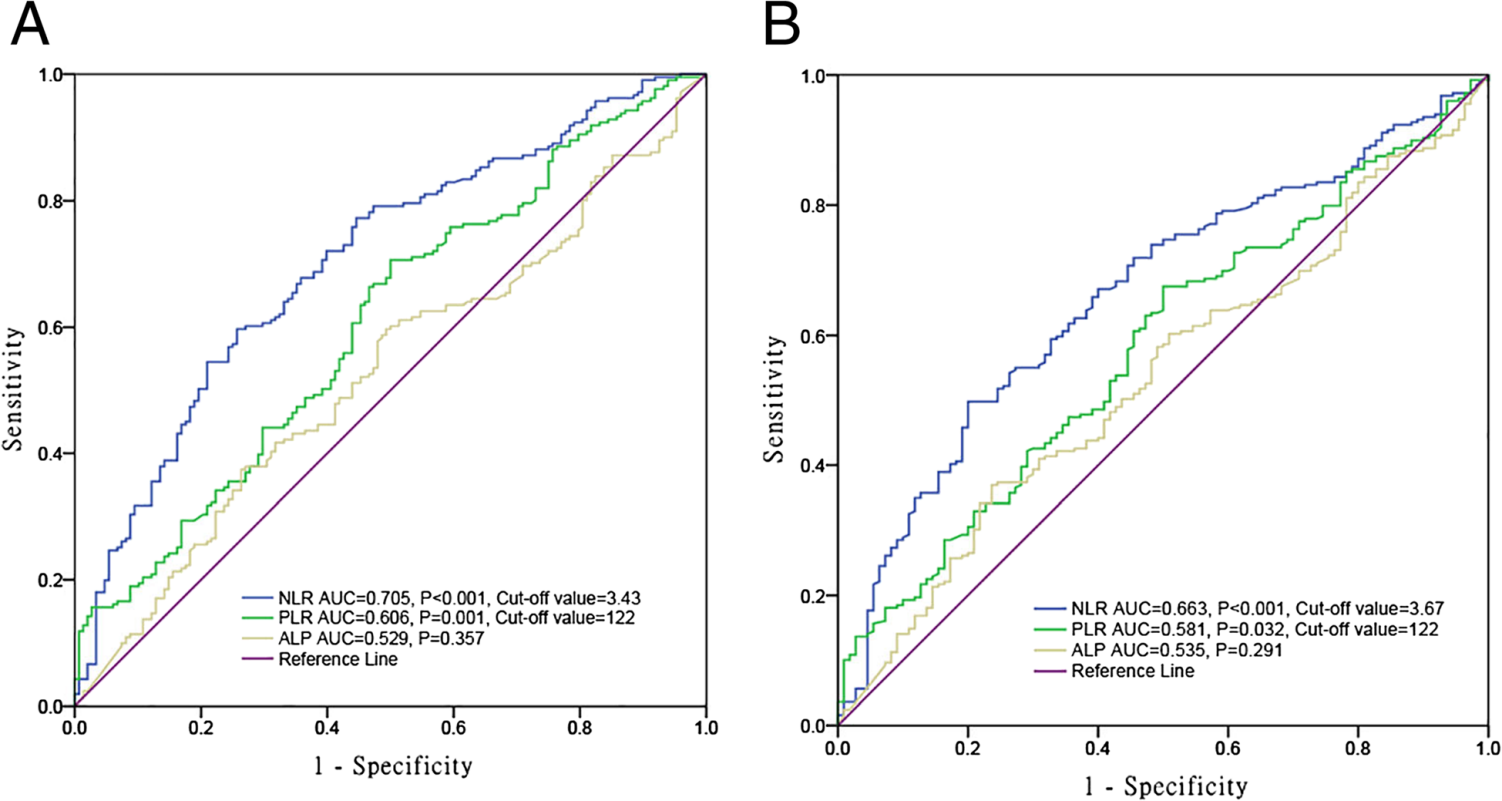
ROC curves for NLR, PLR, and ALP. **a** Overall survival (cutoff value of NLR, 3.43; PLR, 122). **b** Progression-free survival (cutoff value of NLR, 3.67; PLR, 122)

When OS was employed as an end point for NLR and PLR, the areas under the curve (AUC) for NLR, PLR, and ALP were 0.705 (*P* < 0.001), 0.606 (*P* = 0.001), and 0.529 (*P* = 0.357), respectively (Fig. 1a). Subsequently, ALP was excluded due to its small AUC (*P* > 0.05). The optimal cutoff values were 3.43 for NLR (sensitivity, 59.7 %; specificity, 74.3 %) and 122 for PLR (sensitivity, 70.6 %; specificity, 50.0 %). Similarly, using PFS as an end point for them, the cutoff values of NLR and PLR were 3.67 (sensitivity, 49.8 %; specificity, 80.0 %) and 122 (sensitivity, 44.6 %; specificity, 66.4 %), respectively (Fig. 1b). All patients were divided into two groups with the high groups that are greater than or equal to the cutoff values (HNLR and HPLR) and the low groups less than the cutoff values (LNLR and LPLR) on the basis of the optimal cutoff values.

### The associations of NLR and PLR with clinicopathological features

To investigate the associations of NLR and PLR with clinicopathologic features of osteosarcoma patients, comparisons between the high and low groups for NLR and PLR was carried out (Table 1). Our results indicated that advanced stage and metastasis at diagnosis were significantly associated with the high NLR and PLR groups. Elevated ALP and older age were closely associated with the high NLR and PLR, respectively.

### Prognostic factors for OS and PFS

**Fig. 2:**
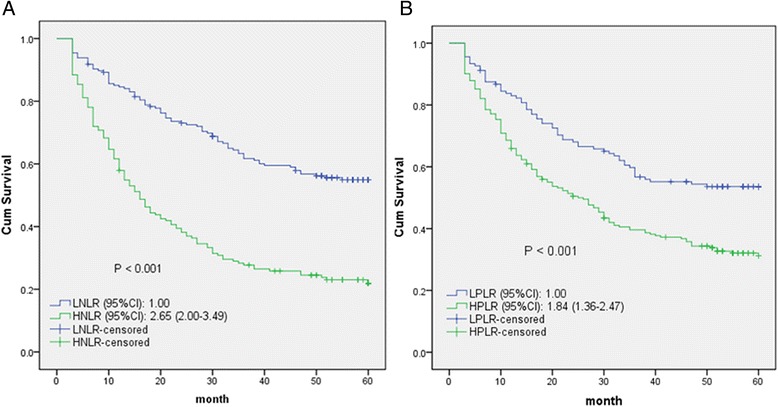
Kaplan-Meier curves for overall survival probability according to NLR and PLR levels (**a**, **b**)

**Fig. 3:**
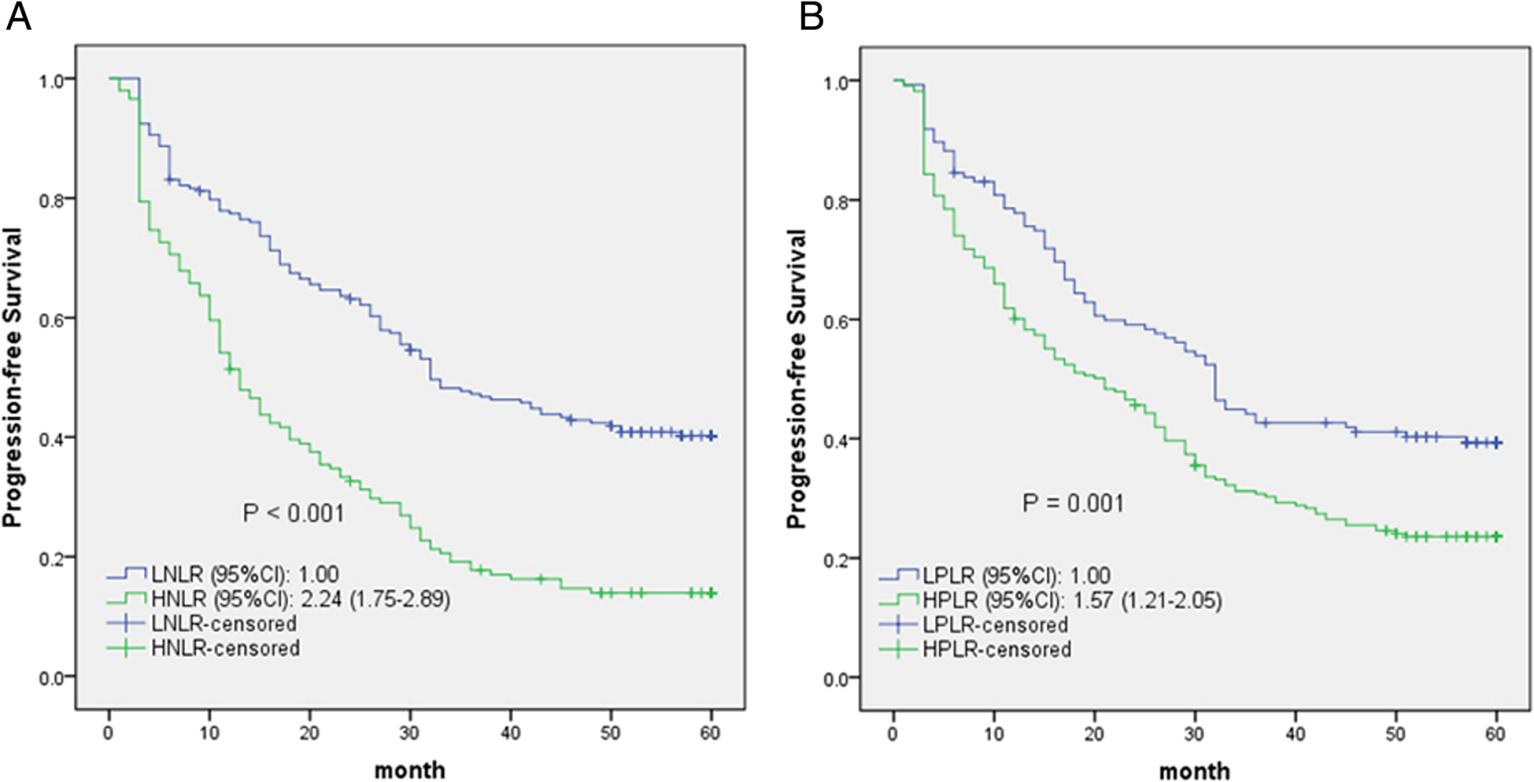
Kaplan-Meier curves for progression-free survival probability according to NLR and PLR levels (**a**, **b**)

The 5-year OS rates of the HNLR and HPLR groups were significantly lower than those of the LNLR and LPLR groups (*P* < 0.001; Fig. 2a, b). Similarly, osteosarcoma patients with HNLR and HPLR had poorer PFS than those with LNLR and LPLR (*P* ≤ 0.001; Fig. 3a, b).

Subsequently, the multivariate analyses of OS and PFS were performed including age, sex, and clinical variables with univariate log-rank *P* < 0.05: clinical stage, metastasis at diagnosis, NLR, and PLR. HNLR, clinical stage, and metastasis emerged as markers for shorter OS and PFS (Table 2). However, HPLR was not an independent indicator for both OS and PFS.

**Table 2:** Univariate and multivariate analyses of clinical parameters for the prediction of overall and progression-free survival

Clinical parameters	Overall survival				Progression-free survival			
	Univariate analysis		Multivariate analysis		Univariate analysis		Multivariate analysis	
	HR	*P*	Adjusted HR (95 % CI)	*P*	HR	*P*	Adjusted HR (95 % CI)	*P*
Age (year)								
≦50	1.00		1.00		1.00		1.00	
>50	1.09	0.540	1.08 (0.82–1.41)	0.598	1.10	0.436	1.08 (0.84–1.39)	0.543
Sex								
Male	1.00		1.00		1.00		1.00	
Female	0.93	0.634	1.06 (0.78–1.44)	0.695	0.97	0.844	1.02 (0.77–1.35)	0.889
Tumor location								
Tibia/femur	1.00				1.00			
Elsewhere	0.99	0.963			0.89	0.378		
Pathological fracture								
Yes	1.00				1.00			
No	0.81	0.275			0.98	0.887		
Tumor size					1.00			
<8 cm	1.00				1.04	0.754		
≥8 cm	1.09	0.524						
ALP								
Normal	1.00				1.00			
Elevated	1.28	0.094			1.30	0.055		
Clinical stage^a^								
I–II	1.00		1.00		1.00		1.00	
III	4.63	0.000	2.49 (1.66–3.72)	0.000	3.32	0.000	2.24 (1.59–3.15)	0.000
Metastasis at diagnosis								
Absent	1.00		1.00		1.00		1.00	
Present	5.09	0.000	2.43 (1.69–3.49)	0.000	3.42	0.000	1.72 (1.23–2.40)	0.002
Post-chemotherapy								
Yes	1.00				1.00			
No	1.02	0.865			1.05	0.687		
NLR								
Low NLR	1.00		1.00		1.00		1.00	
High NLR	2.65	0.000	1.80 (1.35–2.41)	0.000	2.24	0.000	1.65 (1.26–2.15)	0.000
PLR								
Low PLR	1.00		1.00		1.00		1.00	
High PLR	1.84	0.000	1.27 (0.93–1.73)	0.136	1.57	0.001	1.17 (0.88–1.55)	0.273

*ALP* alkaline phosphatase, *WBC* white blood cell, *NLR* neutrophil-to-lymphocyte ratio, *PLR* platelet-to-lymphocyte ratio, *HR* hazard ratio, *CI* confidence interval

^a^Clinical stage according to Enneking surgical stage

### Prognostic nomogram for OS

**Fig. 4:**
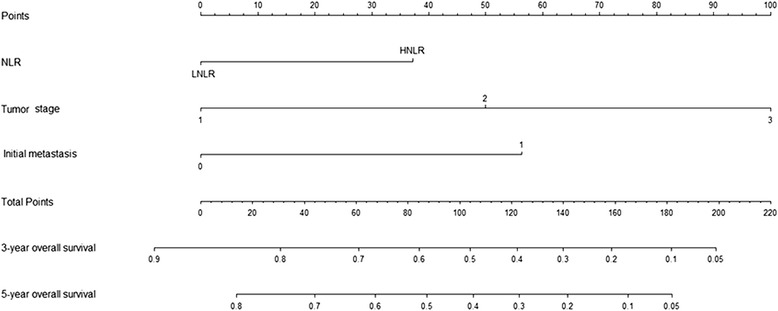
Post-operative nomogram with NLR and significant clinicopathologic characteristics predicted the probability of osteosarcoma for overall survival

To further predict the survival of osteosarcoma patients after surgical resection, a predictive model was constructed by Cox regression model analysis using all the significant independent risk factors for OS (Fig. 4). It can predict the probability of death of osteosarcoma within 3 or 5 years after treatment, assuming the patient does not die of another cause first. The *c*-index for OS prediction was 0.829.

## Discussion

We investigated two factors which have reflected a systemic inflammatory response. The pre-treatments HNLR and HPLR in the peripheral blood of osteosarcoma patients were significantly associated with tumor progression and poor prognosis at post-operation. Despite substantial progress in the understanding of the association between inflammatory biomarkers and prognosis of various cancers [11–13], the influence of inflammatory markers on the prognosis of osteosarcoma patients remains confused. Herein, this study was the first attempt to evaluate the prognosis of patients with osteosarcoma based on inflammatory biomarkers in the peripheral blood and to construct a predictive model to improve the predictive accuracy. Because the peripheral blood count test is routinely performed without the need for additional effort in all patients with cancer, it is a simple, inexpensive, and reproducible parameter of the inflammatory response as well as an indicator of prognosis. Interestingly, only NLR could be considered as an independent indicator for OS and PFS in multivariate analysis.

Rudolf Virchow first reported that “lymphoreticular infiltrate” reflected the origin of tumor at the sites of chronic inflammation [14]. Over the past decades, the emerging evidence verified Virchow’s hypothesis revealing the influence of inflammatory microenvironment on tumor. The inflammatory reaction, which is implicated in repair of tissue damage due to tumors, is an indispensible factor in the tumor cell microenvironment [15, 16]. Thus, inflammatory cells are involved in cell proliferation, invasion, migration, angiogenesis, and metastasis. Meanwhile, tumors can result from inflammatory sites, possibly through the recruitment of inflammatory cells, chemokines, and cytokines. Therefore, the adaptive immune system is converted, and this inflammatory response reactivates tumor development and progression. The inflammatory response could result in neutrophilia, leukocytosis, thrombocytosis, and lymphocytopenia [17]. The platelets may increase angiogenesis and release growth factors to participate in the inflammatory reaction [18]. The lymphocyte response plays a critical role in the suppression of tumor progression [19]. The possible mechanisms underlying neutrophilia in progression and metastasis have release of reactive oxygen species or nitric oxide and remodeling of the extracellular matrix [20]. Hence, more deep understanding of the links between inflammation and tumor contributes to the treatment and prevention of tumor. Several markers have been reported to reflect the association of inflammation and tumor, such as interferon-gamma/interleukin-4 ratio [21] and inflammation-based prognostic score based on CRP and albumin levels [22]. The systemic inflammatory markers (NLR and PLR) may be also regarded as potential prognostic factors for various types of tumors. Azap et al. used pre-treatment NLR and PLR as prognostic indicators of long-term mortality in patients with breast cancer [23]. Deng et al. reported that pre-operative NLR was a superior independent prognostic factor for cancer-specific survival in surgical patients with gastric cancer [11]. He et al. compared NLR to PLR as an adverse prognostic factor in metastatic colorectal cancer and suggested that NLR was superior to PLR [24].

Little evidence has shown that NLR and PLR are associated with prognosis in osteosarcoma. Our study was the first attempt to evaluate the impact of NLR and PLR on prognosis of 359 osteosarcoma patients and establish a predictive model to improve the predictive accuracy for 3-year and 5-year overall survival. Similar to other results in various cancers, pre-treatment NLR and PLR were significantly associated with poor prognosis. Interestingly, only NLR could be considered as an independent prognostic factor for both OS and PFS in patients with osteosarcoma. Our constructed nomogram performed well in the prediction of overall survival (*c*-index 0.829). These data supported that the nomogram could better predict prognosis in osteosarcoma patients at pre-treatment.

Some limitations of our study should be acknowledged. Firstly, the study was a retrospective design, with a small population size of 359 patients, which resulted in no significant correlation between chemotherapy and clinical prognosis. Secondly, the peripheral blood findings were not compared with findings of peritumoral inflammation in the primary tumor tissue. Nevertheless, the data in the peripheral blood provided a novel horizon to understand the roles of NLR and PLR in the development and progression of osteosarcoma. Finally, there were some heterogeneities in the treatment of osteosarcoma patients, which resulted in different clinical prognosis. Hence, further studies are necessary to illuminate the relationship between inflammatory biomarkers and prognosis in patients with osteosarcoma.

## Conclusions

The pre-treatment NLR and PLR are associated with survival in patients with osteosarcoma. NLR is a better predictor of OS and PFS than PLR. Integrate NLR and PLR and the prognostic nomogram may be used to evaluate clinical prognosis and offer optimal therapeutic strategy. In the future, a well-designed study of NLR, PLR, and other promising indicators should be performed to identify the underlying mechanism.
